# Epidemiology, Risk Factors and Outcome Due to Multidrug Resistant Organisms in Paediatric Liver Transplant Patients in the Era of Antimicrobial Stewardship and Screening

**DOI:** 10.3390/antibiotics11030387

**Published:** 2022-03-15

**Authors:** Anita Verma, Sunitha Vimalesvaran, Anil Dhawan

**Affiliations:** 1 Department of Infection Sciences, King’s College Hospital, London SE5 9RS, UK; 2 Department of Paediatric Gastroenterology, Hepatology and Nutrition, King’s College Hospital, London SE5 9RS, UK; sunitha.vimalesvaran@nhs.net (S.V.); anil.dhawan@nhs.net (A.D.)

**Keywords:** liver transplantation, paediatric, multidrug resistant organisms, antimicrobial stewardship

## Abstract

(1) Background: Multidrug-resistant organisms (MDRO) are a growing problem in liver transplant recipients (LTR), associated with high morbidity and mortality. We reviewed the impact of antimicrobial stewardship (AMS) and active screening of MDRO on the epidemiology and outcomes in paediatric LTR. (2) Methods: Single-centre retrospective review of paediatric LTR from January 2017 to December 2018. (3) Results: Ninety-six children were included; 32 (33%) patients were colonised with ≥1 MDRO and 22 (23%) patients had MDRO infections. Median (IQR) duration for start of infection was 9.5 (1.8–16.0) days. Colonisation rate with Gram-positive MDRO was 15.6%, with infection rate of 6.2%; majority due to Vancomycin-Resistant Enterococcus faecium (VRE). Colonisation with Gram-negative MDRO was 27.0%, with infection rate of 16.6%; majority due to extended-spectrum β-lactamase producing *Enterobacteriaceae*. Colonisation and infection rate due to Carbapenem-resistant *Enterobacteriaceae* was 6% and 3%, respectively, during screening and AMS, compared to historical control of 25% and 30%, respectively, without screening and AMS. There was significant reduction in VRE and CRE infection during AMS period in comparison to historical control. Pre-transplant risk factors including bacterial infections pre-transplant (*p* < 0.01), diagnosis of biliary atresia (*p* = 0.03), exposure to antibiotics (*p* < 0.01), EBV viraemia (*p* = 0.01), and auxiliary transplantation (*p* < 0.01) were associated with post-transplant MDRO infections. Patients with MDRO infections had longer length of hospital and paediatric intensive care unit stay days (*p* < 0.01) but associated with no mortality. (4) Conclusions: Our results demonstrate low incidence of colonisation and infections with MDRO, which were associated with high morbidity but no mortality in paediatric LTR. There was significant reduction in MRSA, VRE, and CRE during AMS period compared to pre-AMS era. Some risk factors are unavoidable but antibiotic overuse, early initiation of appropriate antibiotic therapy and effective infection prevention strategies can be monitored with multifaceted approach of AMS and screening of MDRO. With limited therapeutic options for MDRO and efficacy data of newer antibiotics in paediatric LTR, robust infection control practices are of paramount importance.

## 1. Introduction

Despite the advancements in surgical techniques, immunosuppressive therapies, and improved post-operative care, bacterial infections remain the most important complication amongst paediatric liver transplant recipients (LTR) [1,2,3,4,5,6]. The overall incidence of bacterial infections varies from centre to centre, with studies reporting an incidence of 25 to 68% [1,2,3,4,7,8]. Over the last two decades, the rates of multidrug resistant organisms (MDRO) including Methicillin Resistant *Staphylococcus aureus* (MRSA), Vancomycin-resistant *Enterococci* (VRE) and Gram-negative multidrug resistant organisms (GN-MDRO) bacteria have continued to rise and has been increasingly reported in adult and paediatric solid organ transplant (SOT) recipients [4,5,9,10,11]. These MDRO, especially VRE and GN-MDRO, are associated with high rates of morbidity and mortality [12,13,14,15]. Amongst the GN-MDRO, Carbapenem-resistant *Enterobacteriaceae* (CRE), particularly Carbapenemase producing *Enterobacteriaceae* (CPE), are the most worrying organisms, because of emerging pan-drug resistance and it has become the leading cause of morbidity and mortality among LTR [16].

Paediatric LTR are potentially at higher risk for colonisation and infection with MDRO due to the increased exposure to antibiotics and the hospital environment from a young age [10]. Furthermore, these pathogens are often associated with major outbreaks in paediatric LTR [17].

The World Health Organisations and other organisations have highlighted antimicrobial stewardship (AMS) programmes, screening, and infection control as the key instruments to combat the emergence of MDRO and their global spread [18,19]. These have a significant impact on antimicrobial use, costs to healthcare services, and reduction in antimicrobial resistance [20].

Our expertise and relatively high-volume of paediatric liver transplantation in the United Kingdom has provided us with a breadth of patients to review our experience of MDRO in LTR. We describe the epidemiology, risk factors and outcome with MDRO in paediatric LTR and multi-visceral transplant recipients during screening and AMS period.

## 2. Results

### 2.1. Baseline Characteristics of Patients

The 96 children with acute or chronic liver disease who underwent liver (90/96) or multi-visceral (6/96) transplantation over the two-year period were included. The most common indication for transplantation was chronic liver disease secondary to biliary atresia. Table 1 shows baseline characteristics, categorised by their infection status.

### 2.2. Colonisation and Infection with MDRO

Overall, 41% of patients had either colonisation and/or infection over the 2-year period. Of these, 33% patients were colonised with MDRO (Table 2). The colonisation rate with MRSA, CPE, and non-CP-CRE was 10.4%, 6.2%, and 4.2%, respectively (Table 2); all detected before transplantation. Overall, 23% of patients had confirmed MDRO infections post-transplantation (Table 3), with over 95% being colonised pre-transplant. Most infections were due to GN-MDRO (16.6%). Predominant Gram-positive MDRO (GP-MDRO) infections were with VRE (4.2%) and GN-MDRO with ESBL-E (7.3%), respectively (Table 2). Infections with CPE and non-lactose fermenter were 3.1% and 4.1%, respectively. Blood stream infections (BSI), intra-abdominal infections (IAI) or surgical site infections (SSI) were most common. Median (IQR) duration for start of infection after transplantation was 9.5 (1.8–16.0) days (Table 4).

### 2.3. Antibiotic Use

A significantly higher rate of broad-spectrum antibiotics—meropenam and linezolid—were used in patients with MDRO infections (Table 4). All patients received piperacillin-tazobactam prophylaxis post-transplantation, with duration of treatment ranging from one to five days. Only one patient had an isolate of CPE that was pan-drug resistant, and this patient was treated successfully with source control and combination antibiotic therapy. Vancomycin was used equally in both group.

### 2.4. Risk Factors for MDRO Infection

Patients with MDRO infections were of a younger age, with a significantly shorter waiting time from pre-transplant assessment to the transplant (Table 1). There was a statistically higher incidence of infections (*p* = 0.03) in patients with primary diagnosis of biliary atresia and in those who had Gram-negative bacterial infections (*p* < 0.01) before transplant. Group 2 patients received significantly more antibiotics (*p* < 0.01) prior to transplantation (Table 1).

Patients with MDRO infections had a significantly longer stay in the paediatric intensive care unit (PICU) (*p* < 0.01) and overall hospital stay (*p* < 0.01). The significant risk factors for MDRO infections were; auxiliary transplant (*p* < 0.01), EBV viraemia with a viral load of >50,000 DNA copies/mL (*p* = 0.01) [21] and prior colonisation with MDRO (*p* < 0.01) (Table 4).

Multivariate analysis of significant factors in univariate analysis showed LOHS and colonisation with MDRO, high viral load of EBV and infection with Gram-negative organisms were independent risk factors for MDRO infections (Table 5).

### 2.5. Impact on Mortality

**Figure 1:**
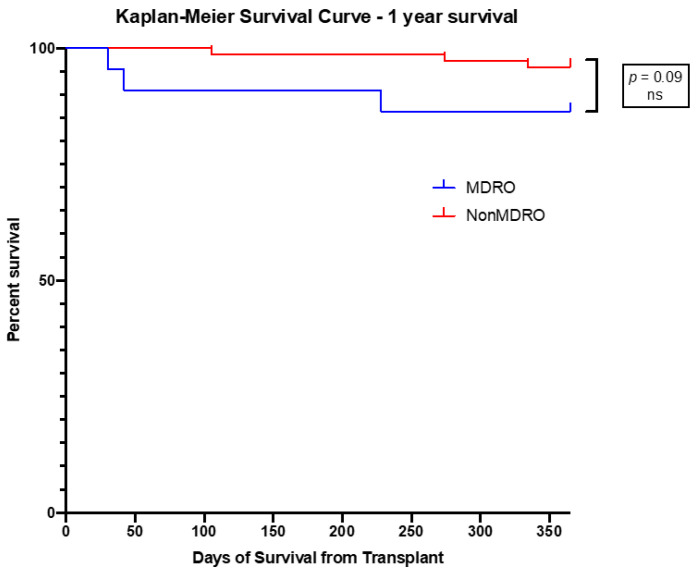
Kaplan–Meier Survival Curve at 1-year of survival of patients with MDRO infections compared to those with no MDRO infection. No significant difference in survival was found.

Survival rates at 30 days, 90 days and 1-year were not statistically different between the two groups (Table 4, Figure 1). None of the patients in Group 2 died of MDRO infections.

## 3. Discussion

To the best of our knowledge, this is the first report to describe the epidemiology, risk factors of MDRO and outcomes in a large cohort of paediatric LTR in the setting of robust AMS and screening.

The incidence of MDRO infections has been increasing among LTR in the last decade [5,12,15,16,22,23]. We report a relatively lower prevalence of MDRO colonisation of 33% in our cohort of patients in contrast to 62%, 48%, and 45% reported by Phichaphop et al., Alcamo et al., and Beranger et al., respectively, in paediatric LTR [4,5,10]. Colonisation and infection rate with GP-MDRO was low compared to GN-MDRO in our centre. GP-MDRO, such as VRE, have been shown in previous studies to have a significant role in paediatric LTR [11]. Overall infection rates with GP-MDRO was only 6%, in contrast to 49% reported by other centres for paediatric LTR [2]. Although we had a high rate of colonisation with MRSA, we observed a low infection rate. The likely explanation for this is early detection of MRSA carrier by screening, early isolation, followed by decolonisation, and the use of vancomycin prophylaxis. Although MRSA infections are declining following these measures, the VRE infection rate has not changed much over the years, both in paediatric and adult liver transplant settings in many centres [11,12]. In contrast, we have observed a significant reduction in VRE infection—down to 5% during AMS in comparison to 22% with no AMS in our historical control. The likely cause for this reduction could be due to significant reduction in antibiotic use. Infections with VRE have been shown to be associated with high mortality and can be seen as an indirect indicator for a complicated post-transplant course, especially in the setting of surgical complications or prolonged hospital stay [23]. Moreover, with the advent of newer antibiotics, such as linezolid and daptomycin, the overall landscape of VRE infections is changing.

Similar to other paediatric and adult LTR studies, colonisation and infections with GN-MDRO was predominant in our cohort of patients [4,5,10]. However, the incidence of GN-MDRO infection of 16% was significantly lower in our centre in contrast to more than 40% reported in other paediatric and adult LTR [5,10,24]. Among GN-MDRO, ESBL-E is the most common cause of Gram-negative bacterial infections, but are less concerning because of broader antibiotic choice to treat infections. In contrast, CRE is a growing problem in LTR and their management is challenging due to limited therapeutic options [25]. In our cohort, the exact colonisation rate with CRE was known because of active screening in comparison to ESBL-E. The overall colonisation rate with CRE was relatively low (10%) pre- and post-transplantation during screening, in contrast to 25% in our historical control when no screening was performed [26]. The routine screening of CRE for all patients was considered in our centre because of high number of sporadic cases and outbreaks in different departments. The CRE incidence rate has been reported sporadically in paediatric transplant patients compared to adult studies [5,27]. Approximately 3–13% of SOT recipients in CRE endemic areas develop CRE infections and the 30-day cumulative mortality rate was reported up to 36% in LTR infected with CPE [28]. In contrast, we have a low infection rate and none of our patients died directly due to any of MDRO infections. This lack of significant mortality difference may be related to an active screening and targeted intensive first line antibiotic therapy with subsequent de-escalation approach, when indicated. In addition, the reduced rate of MDRO is due to early detection, complemented by effective isolation and infections control measures, similar to reports in non-transplant adult patients [29]. Treatment of CRE infections can be challenging because of the limited therapeutic options. Despite new novel antibiotics, there are currently no universal agents available to use safely and effectively for CPE or non-CP-CRE. In our cohort, we treated CRE infections successfully, using a combination therapy of high dose meropenem for CRE with minimum inhibitory concentration of <16 µg/mL ± amikacin or colistin.

Transplant centres, such as ours, that are home to a diverse patient population from across the United Kingdom and internationally, should emphasize the need for active screening and effective infection control practices. The majority of our patients with CRE and MRSA were positive for colonisation on admission (95%), either acquired in the community or from other local hospitals. Active screening provides the opportunity to identify these organisms early on.

Knowledge of risk factors for MDRO colonisation and infection can further help in AMS by mitigating early source control. Colonisation with MDRO was an independent risk factor for MDRO infections after transplant similar to reports by other studies [30]. Risk factors for colonisation with MDRO are ubiquitous but can be prevented. Decolonisation of MRSA has had a significant impact on reducing MRSA infections [31], but there is a lack of data that decolonisation strategies of ESBL-E, VRE, and CRE are effective in reducing the incidence of colonisation and infection [32]. Therefore, in endemic areas and in cases of outbreaks, active screening for ESBL-E, CRE, VRE, and MRSA should be considered according to available resources, so as to isolate MDRO colonised patients and use infection control precautions in order to prevent the spread of these organisms and, therefore, infections. Prior colonisation with MDRO, especially CRE, has been shown to be associated with worse outcomes but it is not considered to be a contraindication to transplantation [33]. Screening and AMS warrants targeting appropriate prophylactic or empirical therapy during and after transplantation, especially in patients where colonisation status is known for CRE, CPE, and MRSA.

MDRO has been reported to be associated with high mortality of 5–7% in paediatric LTR and 15–60% in adult LTR, in contrast to no mortality in our cohort [1,5,10,12,13]. Several adult and paediatric LTR studies have demonstrated that MDRO infections pre-transplantation affects post-transplant survival [10,22]. GN-MDRO has been particularly associated with a higher morbidity and mortality in children and adult patients before and after transplant in comparison to GP-MDRO [3,11,15]. The likely explanation for no mortality due to MDRO in our cohort could be early targeted broad-spectrum antibiotic therapy and source control.

Some risk factors, such as underlying disease, severity, and exposure to antibiotics because of recurrent infections, are unavoidable. In our cohort, children with a diagnosis of biliary atresia were also statistically more likely to succumb to MDRO infections. This group of patients are exposed to more courses of antibiotics due to recurrent episodes of cholangitis—a known complication after Kasai portoenterostomy predisposing them to MDRO infections. We also demonstrated children were more likely to have MDRO infection when they had a shorter time on the waiting list, indicating that these patients were also more unwell prior to transplantation, necessitating multiple admissions, and earlier transplants. In the UK, transplantation is allocated based on the status of the child. MDRO infection was also significantly linked to prolonged exposure to the healthcare environment post-transplantation and in those requiring multiple admissions.

More than 50% of infections were intra-abdominal; the likely precipitating factors for these infections were prior colonisation and invasive devices. Patients with high EBV viraemia had a significantly higher rate of infection, suggesting a possible immunomodulatory effect of EBV infections in this cohort of patients.

This study is subject to the limitations of a single centre, retrospective review. Due to the retrospective nature of data collection from electronic medical records, we were unable to identify some data, which may be better exemplified in a prospective study design. We only screened for MRSA, CPE, and non-CP-CRE, which may underestimate the incidence of MDRO colonisation with VRE or ESBL-E. Although our data represents a diverse population of patients, MDRO infections may vary across the different institutions and geographical locations. However, we believe our study depicts a well-represented population and will aid in understanding MDRO infections better in the paediatric LTR population. We hope this study serves as a platform for larger, multicentre studies in the future.

Our study exemplifies that MDRO infections are a serious threat in paediatric LTR and, as such, focus should be directed toward the prevention of these infections and antibiotic stewardship. An important consideration would be the optimisation and individualisation of antibiotics to all these patients. AMS does help in the reduction in the use of antibiotics.

## 4. Materials and Methods

### 4.1. Patients and Methods

We performed a retrospective, single-centre review of Gram-positive and Gram-negative MDRO (GP-MDRO, GN-MDRO) colonisation and infections in paediatric patients who underwent liver or multi-visceral transplantation between January 2017 and December 2018 (24 months) at the Paediatric Liver Unit at King’s College Hospital (KCH). KCH is a 1200-bedded tertiary referral teaching hospital with many specialities. The Paediatric Liver Unit provides one of the largest comprehensive clinical liver programmes in the world, with integrated investigation and treatment services for all types of acute and chronic liver diseases, including complex pancreato-biliary pathologies. Our unit supports Europe largest liver and multi-visceral transplantation programme. We admit more than 90% of patients from hospitals based in the United Kingdom and 10% from international centres.

### 4.2. Data Collection

The hospital audit committee approved a retrospective data review of all paediatric patients transplanted during this period. Details of all infections and colonisations with MDRO, antibiotics administered (days used), and outcomes were recorded in a password encrypted data spreadsheet. Other patient information including demographic data, underlying diagnosis, post-transplant complications, rejection episodes, viral infections, length of paediatric intensive care unit (PICU) stay, overall length of hospital stay (LOHS), number of hospital admissions, transplant type, and mortality were taken from electronic patient records, laboratory database, and details were collected in a pre-specified data spreadsheet. Data were obtained for up to 1-year post-transplantation. If there was any uncertainty about the classification of the clinical episode, the information was independently reviewed by an individual who was not involved in the data extraction.

### 4.3. Antimicrobial Stewardship (AMS)

AMS included the routine review of antimicrobials through daily remote notifications of patients on antibiotics by a consultant medical microbiologist with expertise in transplant infectious diseases. Further review was completed during weekly ward rounds and in multi-disciplinary team meetings. All decisions are made in co-ordination with the clinical team. The initial antibiotics were prescribed by the clinical team based on local guidelines. The antibiotic guidelines for transplant patients were based on local epidemiology, antibiotic resistance pattern, prior antibiotic exposure, and other risk factors. All paediatric LTR colonised with MDRO received targeted prophylaxis or treatment of infections, based on antibiotic sensitivities. The routine perioperative antibiotic prophylaxis was with intravenous piperacillin/tazobactam ± vancomycin if colonised with MRSA or linezolid if colonised with VRE ± MRSA and ± amikacin if colonised with CRE. The duration of prophylaxis was 1–5 days based on operative or post-operative complications. The infections due to VRE were treated with intravenous or oral (IV/PO) linezolid or daptomycin and MRSA infection with IV vancomycin. Patients infected with CPE were treated with combination therapy of high-dose Meropenem (only if minimum inhibitory concentration of coliform was <16 μg/mL) + Amikacin or IV Colistin or other combination depending on sensitivities pattern of organisms. Early source control (central line change, biliary stent and abdominal drain removal) was considered in hemodynamically stable patients. Duration and dose of antibiotics were rationalised based on site of infection and other risk factors. MDRO prevalence and local antibiotics guidelines are reviewed annually.

### 4.4. Screening of MDRO

All paediatric LTR patients were subject to routine MRSA screening since 2007 and CRE screening since 2012, on admission and weekly thereafter. For MRSA screening, combined nose, throat, axilla, and groin swabs were used, whilst for CRE rectal or stool swabs were used. Bacteria were identified from culture using VITEK^®^2 (bioMerieux, Inc., Durham, NC, USA) or Matrix-assisted laser desorption/ionization (MALDI). All MDRO antimicrobials susceptibility testing was completed using VITEK^®^2 (bioMerieux, Inc., Durham, NC, USA). All positive ertapenem resistant coliforms were tested for five carbapenemases genes (NDM, VIM, OXA-48, KPC, and IMP) by Cepheid PCR. For infection control management, CRE were classified based on presence or absence of carbapenemases gene, as Carbapenemase producing *Enterobacteriaceae* (CPE) and non-carbapenemase producing carbapenem resistant *Enterobacteriaceae* (non-CP-CRE), respectively. All patients with positive MRSA screen received the eradication protocol with 2% chlorhexidine skin wash and nasal mupirocin application. No decolonisation treatment was used for CRE.

Other MDRO such as VRE, extended-spectrum β-lactamase producing *Enterobacteriaceae* (ESBL-E) and non-lactose fermenters MDRO were identified on routine clinical specimens during pre- and post-transplant septic screen investigations.

### 4.5. Infection Control Measures

All patients with MDRO were isolated in a single room and strict infection control precautions were followed including strict hand hygiene, contact precautions, environmental disinfection, and other organism-specific care bundles according to hospital guidelines. All patients colonised with MDRO were considered for 2–4% chlorhexidine skin wash care.

### 4.6. Definition of MDRO Infections

We looked at microbiologically diagnosed infections only. Infections were classified as MDRO or pan-drug-resistant organisms (PDRO) as per the Centres for Disease Control and Prevention (CDC) guidelines. MDRO was defined as an organism resistant to at least one agent in three or more antimicrobial categories, whilst a PDRO was defined as an organism resistant to agents in all antimicrobial categories [34]. CRE was defined as an isolate of *Enterobacteriaceae* resistant to at least one carbapenem.

An infection was defined according to the National Institute of Clinical Excellence (NICE) as the isolation of the pathogen from sterile body fluid, in the presence of signs or symptoms of infection or changes on radiological imaging. Colonisation was categorized as the presence of an organism in a non-sterile specimen, with no signs or symptoms of infections [35].

Urinary tract infection (UTI) was defined as the presence of pus cells and more than 50,000 colony-forming units of a single MDRO identified in a specimen obtained via sterile catheterization in the presence of signs and symptoms. Respiratory tract infection (RTI) was based on clinical signs and symptoms and worsening radiological signs in the presence of a MDRO. Surgical site infection (SSI) was defined by presence of MDRO from a surgical site wound, with consistent clinical signs of infection. Intra-abdominal infection (IAI) was based on clinical signs and symptoms and positive MDRO from an abdominal fluid sample. Blood stream infection (BSI) was based on isolation of MDRO from peripheral venous line. Whereas line related BSI, was defined as isolation of MDRO from a central line and peripheral line [36].

The incidence of MDRO was compared to historical data from our centre for VRE in 2001 and for CRE in 2012; a year before surveillance of CRE commenced [11,17]. During this period, only MRSA screening was conducted.

### 4.7. Statistical Analysis

A pre-specified anonymised dataset fulfilling data protection regulation (GDPR) compliance was prepared for statistical analysis. The cohort was stratified into two groups based on infection status: Group 1—no MDRO infection; Group 2—one or more MDRO infections. Descriptive statistics were performed for demographic data, types of infection and site, and post-transplant data. The incidence of MDRO was calculated, and risk factors were analysed. Risk factors included pre-transplant and post-transplant factors. Categorical variables were described using frequencies and percentages, whilst median and interquartile ranges were used to describe continuous variables. Fisher exact test was used to evaluate categorical variables, whilst the Mann–Whitney test was used for continuous data. Statistical significance was defined as a *p*-value of less than or equal to 0.05. Univariate and multivariate analysis was conducted for significant independent pre- and post-transplant risk factors. Statistical analysis was performed using GraphPad Software version 4.0 (San Diego, CA, USA).

## 5. Conclusions

In summary, our results demonstrate low incidence of colonisation and infections due to MDRO in paediatric LTR. Active screening for CRE and MRSA, has helped us in the rapid identification and early initiation of appropriate antibiotic therapy and effective infection prevention strategies. AMS, including tailored antibiotic treatment for MDRO infections has helped to further reduce resistance and mortality. Risk factors, such as underlying disease, EBV viraemia, and recurrent infections are unavoidable, but antibiotic overuse, and colonisation with MDRO, which are independent risk factors, can be reduced. With limited therapeutic options for CPE or non-CP-CRE and VRE, and especially limited efficacy data of newer antibiotics in paediatric LTR, multifaceted approach of AMS, screening and robust infection control practices are of paramount importance to prevent further spread of these organisms and outbreaks.

## Figures and Tables

**Table 1:** Demographics and risk factors for patients with no MDRO infection (Group 1) compared patients with MDRO infections (Group 2).

Patient Demographics (*n* = 96)	Group 1 (*n* = 74)	Group 2 (*n* = 22)	*p*-Value
Age at time of admission, median (IQR)	3.0 (1.0–10.0)	1.8 (0.9–4.8)	0.12
Sex, *n* (%)			0.48
• Male	37 (50.0)	13 (59.1)	
• Female	37 (50.0)	9 (40.9)	
Time between pre-transplant assessment and transplant date, median (IQR)	91.0 (27.0–214.0)	55.0 (14.0–96.0)	0.04
No. of Inpatient Admissions whilst awaiting transplant, median (IQR)	3.0 (2.0–5.0)	3.0 (1.0–8)	0.85
Underlying diagnosis, *n* (%)			
Acute Liver failure	14 (18.9)	1 (4.5)	0.18
Biliary atresia	29 (39.2)	15 (68.2)	0.03
Metabolic liver disease	5 (6.8)	3 (13.6)	0.38
Others causes	26 (35.1)	3 (13.6)	0.07
Bacterial infection before transplant (MDRO and non-MDRO), *n* (%)	21 (28.3)	18 (81.8)	<0.01
GPO	12 (16.2)	1 (4.5)	0.29
GNO	6 (8.1)	13 (59.1)	<0.01
More than 4 weeks of antibiotic before transplant within 6 months prior to transplant	24 (32.4)	15 (68.2)	<0.01

GPO: gram-positive organism, GNO; Gram-negative organisms, IQR; interquartile range.

**Table 2:** Colonisation and infection pattern of multidrug resistant organisms (MDRO) in paediatric liver transplant recipients.

MDRO (*n* = Colonisation and/or Infection	Colonisation *n* = 32/96, (33%)	Infection *n* = 22/96, (23%)	Infection Types, *n*
Multidrug resistant Gram-positive organisms *n* = 15/96 (15.6%)			
• VRE (*n* = 5)	5 (5.2)	4 (4.2)	BSI:1, UTI:1, IAI:2
• MRSA (*n* = 10)	10 (10.4)	2 (2.0)	SSI:1, HAP:1
Multidrug resistant Gram-negative organisms *n* = 26/96 (27.0%)			
ESBL-E—Extended-spectrum β-lactamase producing Enterobacteriaceae *n* = 9 (9.3%)			
• *Escherichia coli* (*n* = 5)	4 (4.2)	4 (4.2)	BSI:1, IAI:2, UTI:1
• *Klebsiella pneumoniae* (*n* = 4)	3 (3.1)	3 (3.1)	BSI:2, IAI:1
CRE-Carbapenem resistant Enterobacteriaceae *n* = 10 (10.4%)			
(1) CPE—Carbapenemase producing Enterobacteriaceae *n* = 6 (6.2%)			
• VIM—*K pneumoniae* (*n* = 1)	1 (1.0)	1 (1.0)	Cholangitis 1
• VIM—*Citrobacter freundii* (*n* = 3)	3 (3.1)	2 (2.1)	BSI:2
• NDM—*K pneumoniae* (*n* = 2)	2 (2.1)	0 (0.0)	0
(2) Non-CP CRE—Non carbapenemase producing CRE *n* = 4 (4.2%)			
• *Klebsiella pneumoniae* (*n* = 2)	2 (2.1)	2 (2.1)	UTI:1, IAI:1
• *Enterobacter spp* (*n* = 2)	2 (2.1)	0 (0.0)	0
MDR Non lactose fermenters *n*= 7/96 (7.3%)			
*Pseudomonas aeruginosa* (3)	2 (2.1)	2 (2.1)	HAP:2
*Acinetobacter baumnii* (1)	1 (1.0)	0 (0.0)	0
*Stenotrophomonas maltophilia* (2)	2 (2.1)	1 (1.0)	HAP:1
*Elizabethkingia meningoseptica* (1)	1 (1.0)	1 (1.0)	BSI:1

Methicillin-resistant *Staphylococcus aureus*—MRSA, Vancomycin-Resistant Enterococcus faecium-VRE, Colonisation status for VRE and ESBL-E was based on number of patients positive in clinical specimens, e.g., drain fluid, wound swabs, urine, BAL. Blood stream infection; BSI, UTI; urinary tract infection, Intra-abdominal infection: IAI, Hospital acquired pneumonia: HAP; SSI; surgical site infection, 20 patients colonised before transplant.

**Table 3:** MDRO colonisation and infection rates before and after AMS.

MDRO Name	MDRO before AMS		MDRO after AMS	
	Colonisation MDRO/*n* LTR (%)	Infections MDRO/*n* LTR (%)	Colonisation MDRO/*n* LTR (%)	Infections MDRO/*n* LTR (%)
MRSA	41/182 (22.8%)	9/182 (5.6)%	10/96 (10.4%)	2/96 (2%)
VRE	33/182 (18%)	22/182 (12%)	5/96 (5%)	4/96 (4.2%)
ESBL coliforms	NK	26/182 (14.2%)	NK	7/96 (7.3%)
*CPE	21/84 (25%)	13/84 (15.4%)	6/96 (6%)	3/96 (3%)

NK—not known as routine screening not performed. VRE infection data are from 2007 [11]. *CPE—CPE colonisation and infection rate is before AMS period September 2012–December 2013.

**Table 4:** Risk factors for MDRO in post-transplantation (Group 1 vs. Group 2).

Post-Transplant Data (*n* = 96)	Group 1 (*n* = 74)	Group 2 (*n* = 22)	*p*-Value
Type of Transplant			
• Liver *n* (%)	72 (97.3)	18 (81.8)	
• Multi-visceral, *n* (%)	2 (2.7)	4 (18.2)	
Post-Transplant Bacterial Infections			
Days after transplant infection, median (IQR)	0.0 (0.0–12.0)	9.5 (1.8–16.0)	0.79
Length of hospital stay days, median (IQR)	23.0 (17.0–32.0)	76.0 (30.0–94.0)	<0.01
PICU stay days, median (IQR)	3.0 (2.0–7.0)	27.0 (5.5–54.0)	<0.01
Post-transplant antibiotics use, *n* (%)			
• Meropenem	34 (45.9)	18 (85.7)	0.003
• Amikacin	22 (29.7)	15 (71.4)	0.002
• Vancomycin/Teicoplanin	37 (50.0)	12 (57.1)	0.81
• Linezolid	5 (6.8)	6 (28.6)	0.02
Graft type			
• Auxiliary graft	7 (9.5)	14 (63.6)	<0.01
• DCD graft	9 (12.2)	2 (9.1)	0.99
• LLL graft	40 (54.1)	15 (68.2)	0.33
Post-Transplant EBV, CMV and other viral infections			
EBV Viraemia			
>50,000 DNA copies/mL, *n* (%)	13 (17.6)	10 (45.5)	0.01
<50,000 DNA copies/mL, *n* (%)	30 (40.5)	5 (22.7)	0.141
Chronic EBV viraemia > 6 month	32 (43.2)	10 (45.5)	0.99
CMV status high risk (Donor seropositive, recipient seronegative), *n* (%)	20 (27.0)	7 (31.8)	0.79
CMV infection, *n* (%)	26 (35.1)	8 (36.4)	0.99
Non-EBV and CMV viral infections	24 (32.4)	11 (50.0)	0.21
Post-Transplant complications			
Colonisation with MDRO, *n* (%)	11 (14.9)	21 (95.5)	<0.01
Biliary complications, *n* (%)	8 (10.8)	6 (28.6)	0.08
Bowel Perforation, *n* (%)	3 (4.1)	2 (9.5)	0.32
Re-transplant, *n* (%)	3 (4.1)	3 (14.3)	0.13
Graft rejection	43 (58.1)	16 (72.7)	0.32
Overall Mortality, *n* (%)	3 (4.1)	3 (14.3)	0.13
• 30 days Mortality, *n* (%)	1 (1.4)	0 (0.0)	>0.99
• 90 days Mortality, *n* (%)	1 (1.4)	0 (0.0)	>0.99
• 1-year Mortality, *n* (%)	1 (1.4)	3 (14.3)	0.09

Abbreviations: PICU; paediatric intensive care unit, DCD; donation after circulatory death, LLL; left lateral lobe, EBV; Ebstein–Barr virus, CMV; cytomegalovirus, DNA; deoxyribonucleic acid.

**Table 5:** Multivariate analysis.

	MVA, *p* Value
LOHS and Colonisation	0.0001
High EBV and GNO	0.008
BA and Time to transplant	0.166
Graft type (auxiliary, High EBV and GNO)	0.281

Abbreviations: LOHS; length of hospital stay, EBV; Ebstein–Barr virus, GNO; Gram-negative organism, BA; biliary atresia.

## Data Availability

The data presented in this study are available on request from the corresponding author.
